# Editorials

**Published:** 1897-03

**Authors:** 


THE
### HOMŒOPATHIC PHYSICIAN,

A MONTHLY JOURNAL OF

HOMŒOPATHIC MATERIA MEDICA AND CLINICAL MEDICINE.

"If our school ever gives up the strict inductive method of Hahnemann, we are lost, and deserve only to be mentioned as a caricature in the history of medicine."—CONSTANTINE HERING.

Vol. XVII. MARCH, 1897. No. 3.

### EDITORIALS.

MEMBERS OF THE INTERNATIONAL HAHNEMANNIAN ASSOCIATION will please take notice that the editor of this journal is Chairman of the Bureau of Materia Medica.

It is very desirable to have a good showing of this bureau at the next annual meeting, to be held in June next. Materia medica is the great weapon of our cause, and should be developed more than any other subject with which we have to deal. The best work of the annual meeting should therefore be in the line of pathogenesis of drugs. Every member who has anything of value upon this subject should send it at once to the editor at his office, 1231 Locust Street, Philadelphia, Pa.

Owing to the great demand upon his time by reason of his practice, as well as the care of the journal, it will be impossible to send a special letter to each of the members. He therefore takes this method of appealing to their interest in the cause.

---

APOLOGY.—The editor regrets that, having been much overworked this month, he has had neither the time nor the energy to write the usual materia medica editorial. These editorials require much time and much mental concentration for their preparation. They would not be published at all if the profession did not care for them. But the commendations received encourage us in their continued production.

